# Predicting Bitcoin Prices Using Machine Learning

**DOI:** 10.3390/e25050777

**Published:** 2023-05-10

**Authors:** Athanasia Dimitriadou, Andros Gregoriou

**Affiliations:** 1 College of Business, Law and Social Sciences, University of Derby, Lonsdale House, Quaker Way, Derby DE1 3HD, UK; 2 School of Business and Law, University of Brighton, Elm House, Lewes Road, Brighton BN2 4AT, UK

**Keywords:** Bitcoin, machine learning, linear support vector machine, random forest

## Abstract

In this paper we predict Bitcoin movements by utilizing a machine-learning framework. We compile a dataset of 24 potential explanatory variables that are often employed in the finance literature. Using daily data from 2nd of December 2014 to July 8th 2019, we build forecasting models that utilize past Bitcoin values, other cryptocurrencies, exchange rates and other macroeconomic variables. Our empirical results suggest that the traditional logistic regression model outperforms the linear support vector machine and the random forest algorithm, reaching an accuracy of 66%. Moreover, based on the results, we provide evidence that points to the rejection of weak form efficiency in the Bitcoin market.

## 1. Introduction

Does Bitcoin respond to financial, cryptocurrency, and macroeconomic shocks? Should Bitcoin follow the efficient market hypothesis? Do the other cryptocurrencies affect the volatility of Bitcoin prices? Bitcoin emerged in 2009 as the world’s first cryptocurrency, attracting new investors due to high returns. This is reflected by the returns of Bitcoin, as quoted on Coinbase, increasing by more than 120% from 2016 to 2017, reaching USD20.000 from USD900 for the purchase of a single Bitcoin token. In early 2017, the market capitalization of Bitcoin grew significantly from around USD18 billion to nearly USD600 billion at the end of that year. As an investment asset, Bitcoin was originally in the retail sector but has now become the benchmark for all other digital currencies that have emerged, such as Ethereum, XRP and Litecoin, among others.

Prior research has compared Bitcoin to gold due to its low correlation with other financial assets [1]. In a similar vein to gold, Bitcoin can be used to hedge against inflation or economic uncertainty, using futures contracts (Bakkt) and unregulated cryptocurrency derivatives exchanges, such as BitMex, Huobi and OKex [2,3]. The motivation behind this is that Bitcoin has a fixed supply, so it does not suffer from the devaluation problem of paper money that occurs through quantitative easing.

Although there are also some studies that focus on predicting stock market price movements, it is important to consider the cryptocurrency market, which, according to Ferreira et al. [4], is characterized by high volatility, no closed trading periods, relatively smaller capitalization, and high market data availability. However, in an efficient market [5], prices of securities in financial markets fully reflect all variable information. Given that the future is unknown, prices should follow a random walk; that is, future changes in stock (security) prices should, for all practical purposes, be unpredictable. In the weak-form efficiency case, future returns cannot be predicted based on past price changes. Therefore, in the long run, one cannot outperform the market by using publicly available information.

However, Bitcoin and other digital assets are not backed by any tangible assets. In general, Bitcoin and other cryptocurrencies are known to react to certain public market announcements [6,7]. In this regard, the cryptocurrencies market is highly efficient, with prices reflecting accessible real-world information almost instantly.

Various types of modeling methodologies have been applied in an attempt to forecast Bitcoin prices. Among the most prominent techniques are: random forest [8], artificial neural networks [9,10], bayesian neural networks [11], and deep learning chaotic neural networks [12]. However, irrational and unexpected factors such as sentiment have been favored more in empirical research on the Bitcoin market [13,14,15]. Kraaijeveld and de Smedt [14] study to what extent public Twitter sentiment can be used to predict price returns for the nine largest cryptocurrencies, including Bitcoin. Nevertheless, some researchers have examined unexpected US monetary policy announcements, considering that these exercize a significant impact on Bitcoin [16], while others establish that there is a connection between cryptocurrencies and news, more broadly through macroeconomics news announcements. Corbet et al. [16] report that positive news after employment and durable good announcements results in a decrease in Bitcoin returns. However, an increase in the percentage of negative news surrounding these announcements is linked with an increase in Bitcoin returns.

Akyildirim et al. [17] focus on the prediction of cryptocurrency returns by collecting the twelve most liquid daily cryptocurrencies using machine-learning classification algorithms, including the support vector machine (SVM), logistic regression models, artificial neutral networks, and random forest. They find an average classification accuracy close to 50% for all these techniques. Finally, they observe that the SVM gives superior and more consistent results compared to those of logistic regression, artificial neural networks, and random forest classification algorithms. Jaquart et al. [18] also apply machine-learning techniques to predict high-frequency (one minute to 60 min) Bitcoin prices over the period 4 March 2019 to 10 December 2019. They discover that all tested models make statistically viable predictions, forecasting the binary market movement with accuracies ranging from 50.9% to 56.0%. Chen et al. [19] apply several machine-learning methods to forecast high-frequency (5-min intervals) Bitcoin prices. The authors collected daily data between 17 July 2017 and 17 January 2018. For daily forecasting, they observe that statistical methods and machine learning achieve 66% and 65% accuracy, respectively, which outperforms benchmark methods.

In our study, we attempt to uncover the potential relationship between cryptocurrencies and other financial variables using a machine-learning framework on weekly data. To accomplish this, we compile a pool of 24 potential regressors based on economic theory and prior research. Using three different techniques, an SVM model with a linear kernel and a random forest algorithm, we examine the directional forecasting performance of our models in comparison to the commonly used logistic regression model. The innovation of our work stems from the application of state-of-art machine-learning methodology and the empirical identification of a relationship between Bitcoin and other cryptocurrencies and macroeconomic variables. We also specifically test the relationship between Bitcoin prices, exchange rates, and interest rates as a possible empirical validation of the Efficient Market Hypothesis (EMH) under a machine-learning framework.

The results of the empirical investigation provide evidence that the returns on Bitcoin are independent of returns on other cryptocurrencies or macroeconomic determinants. This reveals that Bitcoin is a special asset independent of monetary policy or other digital currencies. According to this, investors could be able to utilize Bitcoin as a hedge against regulatory frameworks affecting interest rates and inflation. Given its ability to act as a hedge and its resistance to quantitative easing due to its limited supply, Bitcoin has the potential to flourish and strongly influence alternative investments for several years to come.

The remainder of the paper is organized in the following way: In the next section, we describe the data. Section 3 outlines the methodology that we use in our research. Section 4 reports our empirical findings. Section 5 summarizes and concludes.

## 2. Data

We developed a binary classifier based on SVM to predict the stock price movements of Bitcoin. The data was collected daily from Coinlore.com, a website providing high-frequency cryptocurrency data. The macroeconomic variables and interest rates were obtained from the Federal Reserve Bank of St. Louis (FRED), and the collection of selected exchange rates were acquired from Yahoo finance. The data spans from the 2nd of December 2014 to 8th July 2019. We compiled a dataset of 24 variables, which included the economic policy uncertainty (EPU) index as a factor, given that an increase in the EPU will change investors’ sentiments for the worse, according to Yen and Cheng [20] (Panel A). We included various exchange rates, such as EUR, GBP, JPY, and AUD, against the domestic country’s USD exchange rate to check whether these currencies affect Bitcoin movements (Panel B). We also assembled the main interest rates that were used as benchmarks for the US and the European economy (Panel C). Moreover, following the literature review that attributes Bitcoin’s movements, we considered that other cryptocurrencies [21] could influence Bitcoin’s volatility (Panel D). Finally, in Panel E, we created three different variables: the momentum for each 5, 10 and 15 days from the start of the dataset, giving more information to the model.

Overall, more than 700 observations were collected, but because the stock exchange is closed on weekends and there were many missing values, we applied a filtering process to the data. After we filtered the data, the final sample consisted of 239 observations. Financial returns ($r_{t})=\Delta P_{t}$ − Δ$P_{t-1}$ were calculated with P denoting the closing prices of each variable in our sample. All the variables in our data, along with summary statistics, are displayed in Table 1. The JPY/USD exchange rate and the cryptocurrency Deutsche eMark (DEM), with values of 0.000436 and 0.000019 respectively, appear to have the smallest positive standard deviations that are close to zero. This indicates that these two factors have the lowest volatility. For the target (output), we modeled the return of Bitcoin, using a binary-dependent variable coded as 0 or 1, where 0 indicates that the return of the Bitcoin value is negative (the value decreased from the previous day) and the 1 indicates that the return of the BTC is positive (the value increased from the previous day).

## 3. Methodology

### 3.1. Logistic Regression Model

Undertaking directional forecasting requires that the dependent variable be binary and take two states: 0 or 1, expressing the next negative and positive Bitcoin return values, respectively. The basic drawback of the ordinary least squares (OLS) regression methodology is that the nature of the dependent variable makes OLS regression results irrelevant due to the heteroskedasticity of the estimated errors and the hypothesis violations in the asymptotic efficiency of the estimated coefficients. To solve this issue, we estimated the probability $P_{i}$ = $E\left(y_{i}=1|x_{i}\right)$ = $\frac{e^{x_{i}\beta }}{1+e^{x_{i}\beta }}$ that the dependent variable is equal to 1. Given the conversion of the dependent variable to binary, the logarithm of the probability of being in state 1 to state 0 is obtained from the following equation, which is called the “logit,” where $x_{i}$ is the vector of the independent regressors and β is a vector of the estimated coefficients.

$$L_{i}=L_{n}\left(\frac{P_{i}}{{1-P}_{i}}\right)=x_{i}\beta^{Τ}$$

If the estimated $L_{i}$ is above 1, we classify it as belonging to class 1, while if it is below 1, we classify it in class 0.

### 3.2. Support Vector Machine

**Figure 1:**
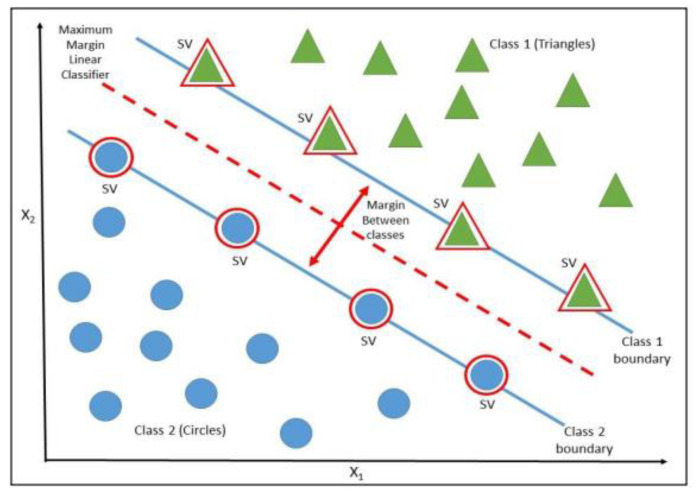
Hyperplane selection and support vectors. The pronounced red circles represent the SVs, thus defining the margins with the dashed lines. The dotted line describes the separating hyperplane.

Data classification and regression tasks usually include the use of the SVM, a supervised machine-learning methodology. It has gained great popularity due to its ability to provide highly accurate prediction results without making a priori assumptions concerning the phenomenon under investigation. Finding the ideal hyperplane that maximizes the distance between the two classes and the highest level of accuracy enables the SVM to classify the data into two classes [22]. A tiny minority of data points known as support vectors (SV) that were found using a minimization technique define the hyperplane. This process is shown visually in Figure 1. In our study, the initial dataset is split into two subsamples: the training set and the testing set. The training step, when the hyperplane is established, receives 80% of the data. The remaining 20% of the total sample is used in the testing set, where the generalization ability of the model is tested on the small part of the dataset that was set aside during the training set.

The hyperplane is defined as:

$$\hat{w}=\sum_{i=1}^{N}a_{i}y_{i}x_{i}\;\hat{b}={\hat{w}}^{T}x_{i}-y_{i},i\in V$$

where V = {i:0 < $y_{i}$ < C} is the set of support vector indices.

The SVM with a linear kernel has become widespread, given that it possesses faster training and classification speeds with significantly fewer memory requirements than nonlinear cores due to the SBM’s compact representation of the decision function. In our research, we also examine the linear kernel where it detects the separating hyperplane in the original dimensional space of the dataset. The mathematical representation of the Radian Basis Function (RBF) kernel is the following:

$$\mathrm{RBF}:\;K\;(x_{1},x_{2})=e^{-{\gamma \left\|x_{1}-x_{2}\right\|}^{2}}$$

**Figure 2:**
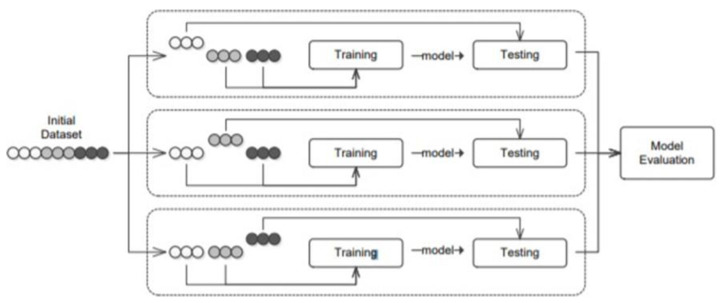
Overview of a 3-fold cross-validation training scheme. It shows that each fold is used as a testing sample, while the remaining folds are used for training the model for each parameter’s value combination.

Over-fitting is a common issue that appears in the training set, where the model “learns” to accurately describe the training data, while giving worse performance to the test set. This concern is described in the literature as the “low bias–high variance” [23,24]. To avoid over-fitting, we use a cross-validation framework, displayed in Figure 2. The initial training set is split into n equal-sized parts. The training step is performed n times, using a different sample for testing, and the rest of the model is repeated in n − 1 parts, each time holding one part for test purposes. This process is reiterated n times with the same set of parameters until all parts of the test process have passed, evaluating the average accuracy of the model performance for that set of hyperparameters in all n parts of the test. Based on our study, we use a 5-fold cross-validation procedure 5 times, applying and evaluating its accuracy on the sample of 20% of the data.

### 3.3. Random Forests

Random forest is an ensemble technique that combines the idea of decision trees with the bootstrapping and aggregating procedure to create a diversified pool of individual regression systems [25]. The random forest algorithm is referred to in the literature by many researchers as a method commonly used to avoid overfitting issues that may arise in decision trees by combining multiple decision trees into a setup called random forest [26,27]. Each tree is constructed from a random set of features where there is a replacement subsample of size n, the same as in the initial dataset. The observations that were not selected in the bootstrapping process form the out-of-bag (OOB) set used for the testing generalization ability of the trained model. To reduce the dependence of the models on the training set, each tree uses a randomly selected subset of the explanatory variables (features). Normally, we use the square root of the total number of features. The system aggregates the classification of each tree and retains the most popular class.

### 3.4. Performance Matrix

Our study uses four separate performance indicators to illustrate how effectively the machine-learning categorization models execute detailed forecasting. The confusion matrix is created as shown in Table 2, where the predictive scores are binary and just one single confusion matrix can analyze it. Each category of the confusion matrix (TN, FN, FP, TP) is evaluated separately. Specifically, the TN expresses the number of predictions that were correctly classified in the negative category, while the FP implies the number of predictions that were incorrectly classified in the positive category. Also, the FN expresses the number of predictions that were incorrectly classified in the negative category, while the TP declares the number of predictions that were correctly classified in the positive category.

Based on the results of the confusion matrix, the following performance metrics are computed to evaluate the models.

(1) $$\mathrm{Recall}=\frac{TP}{TP+FN}$$

(2) $$\mathrm{Accuracy}=\frac{TP+TN}{TP+FN+FP+FN}$$

(3) $$\mathrm{Precision}=\frac{TP}{TP+FN}$$

(4) $$F1-\mathrm{Score}=2\;\times \;\frac{Precision\times Recall}{TPrecision+Recall}$$

All performance metrix ratios range from 0 to 1. In our research, accuracy is the key performance matric to evaluate and compare the machine-learning models, as the models do not have balanced problems between the two classes of the target variable. Accuracy is expressed as the ratio of all the true predictions (positives and negatives) to the total number for all datasets. Moreover, accuracy is considered a significant performance metric in classification problems [28,29]. However, when the dataset has unbalanced data, a high value of accuracy can be a misleading factor since the models tend to choose the majority class, achieving extremely high accuracy (“Accuracy Paradox”) [30].

Precision estimates the ratio of true positives cases among all cases (both true and false), showing how many times our model predicted the positive class, and the numerator counts how many of those classes were actually positive, while the F1-Score is the harmonic mean of precision and sensitivity. Recall is the fraction of the true positive instances (cases) among all the cases (both true and false), reporting all the positive cases. The numerator counts how many of those cases were correctly predicted by our model.

## 4. Empirical Results

Given the scope of this study, we apply a coarse-to-fine grid search scheme on the training set. We can obtain the optimal values of the hyperparameters that maximize the predictive ability of the SVM and random forest models. To accomplish this, we use a 5-fold cross validation process, avoiding overfitting issues. Given the balanced nature of our dataset, the procedure continued to identify the best parameters of the optimal model. The results of the hyperparameters of the SVM model with an RBF kernel are c = 0.0001 and γ = 100, while the optimal hyperparameters for the random forest model that we tested were n-estimators = 75 (total numbers of decision Trees).

However, the generalization ability of the trained model is evaluated using the testing dataset, which includes 239 observations. Results of 96 observations present an upward trend in the Bitcoin’s price, while 143 observations have a negative direction. As a performance matric, we employ four different metrics, recall (sensitivity), accuracy, precision, and F1-Score.

According to Table 3, the random forest and SVM with RBF kernel represent the same accuracy performance of 58%. However, the Logit model achieves a significantly higher predictive performance for all performance metrics. The performance of accuracy gives the highest result of 0.66. This implies that 66% is expressed as true predictions (positive and negative) in the total number for all the data. The precision is likewise the highest (53%) through all metrics. This means that from the cases that the model forecasts an increase in Bitcoin return (true positives + false positives), 53% are actual increased values (true positives), so were correctly anticipated each time the model predicted this category.

## 5. Conclusions

Bitcoin has evolved rapidly over the past decades and is attracting strong attention from investors, who see this as part of the alternative investment space. With this significantly growing attention from the investment community, Bitcoin is an important asset class for researchers and traders alike. The objective of our paper is to construct a model which predicts Bitcoin movements and to investigate whether Bitcoin follows an efficient market hypothesis or a random walk. To achieve this, we collect a large dataset consisting of 24 variables that includes exchange rates, interest rates, macroeconomic variables, another 13 cryptocurrencies, and four auxiliary variables, spanning the period from 2 December 2014 to 8 July 2019. The dataset includes 239 observations (5-days frequency), divided into two subsamples: in-sample and out-of-sample. Two different machine-learning techniques and a traditional regression model are used, namely, logistic regression, the support vector machine and the random forest algorithm, which demonstrate the predictability of the upward or downward price moves. For the machine-learning model, the optimal values of the respective hyperparameters were initially found using five-fold cross-validation and out-of-bag methods to avoid overfitting.

**Figure 3:**
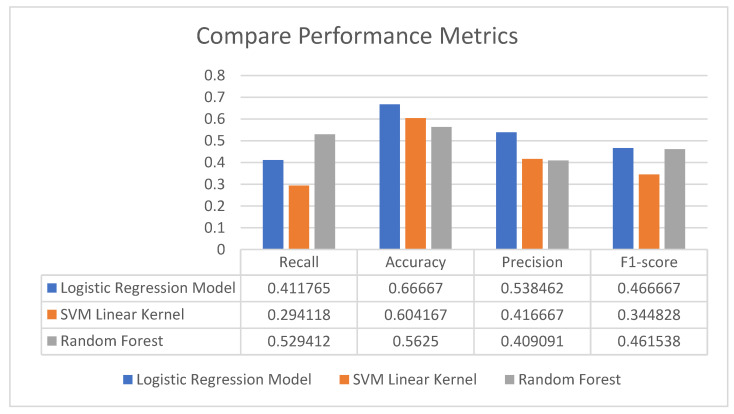
Aggregated results and comparison of proposed methodologies.

Figure 3 summarizes the results of the three forecasting methodologies used. A traditional logit model achieved the best performance (66% accuracy) for Bitcoin movements. However, all the other performance metrics have almost similar and lowest results.

The empirical analysis confirms that the returns of Bitcoin are not affected by the returns of other cryptocurrencies or macroeconomic variables. This implies that Bitcoin is a unique asset that is not related to economic policy or other digital currencies. This suggests that investors can use Bitcoin as a hedge against government policy on inflation and interest rates. Given its hedging qualities and its robustness to quantitative easing due to its fixed supply, Bitcoin has the ability to continue to grow and make an important contribution to alternative investments for years to come.

## Figures and Tables

**Table 1:** Descriptive statistics of 18 cryptocurrencies and exchange rates.

Variables	Name	Std	Mean	Skew	Kurt
**TARGET**	Bitcoin	0.491265	0.401674	0.403676	−1.852619
**Panel A: Macroeconomic Variables**					
**USEPUINDXD**	Economic Policy Uncertainty Index for United States	44.333749	87.418201	1.409511	4.538997
**Panel B: Exchange Rates**					
**EUR/USD**	EUR/USD	0.045994	1.133789	0.455684	−0.134286
**GBP/USD**	GBP/USD	0.106192	1.370556	0.548099	−1.066357
**JPY/USD**	JPY/USD	0.000436	0.008880	0.010883	−0.234481
**AUD/USD**	AUD/USD	0.031169	0.749203	0.203230	−0.407285
**Panel C: Interest Rates**					
**TB3MS**	3-Month Treasury Bill Secondary Market Rate, Discount Basis	44.33288	87.412762	1.409956	4.540290
**DFII10**	Market Yield on U.S. Treasury Securities at 10-Year Constant Maturity	0.414795	2.325826	0.085033	−0.639574
**Panel D: Cryptocurrencies**					
**BTC Real**	Bitcoin Real Price	3783.646	3371.3188	1.320277	1.466443
**DOGE**	Dogecoin	3781.398	3375.7582	1.320594	1.469247
**MAID**	MaidSafeCoin	0.197630	0.195004	1.814227	−0.328242
**XRP**	XRP	0.349752	0.231999	3.025756	13.524215
**NVC**	Novacoin	1.998961	1.882093	1.768265	2.927803
**NMC**	Namecoin	0.937335	0.955977	2.516421	8.185247
**LTC**	Litecoin	60.35639	43.998117	2.018088	4.684777
**GLC**	Goldcoin	0.071203	0.053806	2.404576	7.640745
**DASH**	Dash	218.0163	142.67451	2.471539	6.954688
**DEM**	Deutsche eMark	0.000019	0.000009	3.678487	15.999390
**ABY**	ArtByte	0.005136	0.002809	3.464867	15.451262
**DIME**	Dimecoin	0.011868	0.009364	3.020989	10.973465
**ORB**	Orbitcoin	0.163214	0.139930	2.294509	6.639723
**GRS**	Groestlcoin	0.380965	0.246300	2.019766	4.325836
**Panel E: Momentum Variables**					
**MOM5**	Momentum 5-Days	1.146598	0.246300	0.412184	−0.195015
**MOM10**	Momentum 10-Days	1.591595	3.979079	0.349793	−0.117683
**ΜOΜ15**	Momentum 15-Days	2.102353	6.016736	0.311525	−0.487390

**Table 2:** Classification Results using Confusion Matrix.

		Predicted Label	
		0	1
**Actual**	0	TN	FP
		(True Negatives)	(False Positives)
	1	FN	TP
		(False Negatives)	(True Positives)

**Table 3:** Performance metrics of the three methodologies.

	*Recall*	*Accuracy*	*Precision*	*F1-Score*
***Logistic Regression Model***	0.411765	0.66667	0.538462	0.466667
***SVM Linear Kernel***	0.058824	0.583333	0.200000	0.090909
***Random Forest***	0.588235	0.583333	0.434783	0.500000

Notes: Three different performance metrics evaluated and analyzed using the Logistic Regression model, support vector machine, and random forest technique.
